# Dynamics of SARS-CoV-2 Spike Proteins in Cell Entry: Control Elements in the Amino-Terminal Domains

**DOI:** 10.1128/mBio.01590-21

**Published:** 2021-08-03

**Authors:** Enya Qing, Tom Kicmal, Binod Kumar, Grant M. Hawkins, Emily Timm, Stanley Perlman, Tom Gallagher

**Affiliations:** a Department of Microbiology and Immunology, Loyola University Chicago, Maywood, Illinois, USA; b Department of Microbiology and Immunology, University of Iowa, Iowa City, Iowa, USA; Columbia University Medical College

**Keywords:** SARS-CoV-2, coronavirus, coronavirus spike protein, membrane fusion, virus entry, virus receptors

## Abstract

Selective pressures drive adaptive changes in the coronavirus spike proteins directing virus-cell entry. These changes are concentrated in the amino-terminal domains (NTDs) and the receptor-binding domains (RBDs) of complex modular spike protein trimers. The impact of this hypervariability on virus entry is often unclear, particularly with respect to sarbecovirus NTD variations. Therefore, we constructed indels and substitutions within hypervariable NTD regions and used severe acute respiratory syndrome coronavirus 2 (SARS-CoV-2) virus-like particles and quantitative virus-cell entry assays to elucidate spike structures controlling this initial infection stage. We identified NTD variations that increased SARS-CoV-2 spike protein-mediated membrane fusion and cell entry. Increased cell entry correlated with greater presentation of RBDs to ACE2 receptors. This revealed a significant allosteric effect, in that changes within the NTDs can orient RBDs for effective virus-cell binding. Yet, those NTD changes elevating receptor binding and membrane fusion also reduced interdomain associations, leaving spikes on virus-like particles susceptible to irreversible inactivation. These findings parallel those obtained decades ago, in which comparisons of murine coronavirus spike protein variants established inverse relationships between membrane fusion potential and virus stability. Considerable hypervariability in the SARS-CoV-2 spike protein NTDs also appear to be driven by counterbalancing pressures for effective virus-cell entry and durable extracellular virus infectivity. These forces may selectively amplify SARS-CoV-2 variants of concern.

## INTRODUCTION

As with other human coronaviruses (CoVs), severe acute respiratory syndrome CoV 2 (SARS-CoV-2) emerged in humans from infected animals (1, 2). Its subsequent efficient airborne transmission, frequently proceeding prior to clinical disease, accounted for its remarkably rapid pandemic spread. With expansion, the virus adapted to its new human hosts. The first adaptive variations were identified within months after human infections were recognized (3, 4). These variant viruses exhibited increased transmissibility, such that by late 2020, less than 1 year into the CoV disease 2019 (COVID-19) pandemic, SARS-CoV-2 variants dominated the human-circulating virus population. The current SARS-CoV-2 viruses are now classified onto several variants of concern (VOCs) due to their high transmissibility, their potential for bypassing natural infection and vaccine-induced immunity, and their possibly increased virulence (5).

The concerning SARS-CoV-2 variations are within the viral spike (S) proteins. Intercellular and human-to-human transmission requires S proteins, as they direct virus entry into oro-nasal, airway, and alveolar epithelial cells (6–8). The S proteins are complex ∼500-kDa homo-trimers, operating as molecular machines that bind viruses to target cells and catalyze the fusion of virus and cell membranes. These functions are executed by several S protein domains that are arranged into metastable “prefusion” configurations. Multidomain S1 portions that are distal from the virion envelopes bind to attachment factors and bona fide protein receptors (9, 10). Binding reorients S1 relative to virion-proximal S2 portions (11, 12), allowing S2 to extend, capture cell membranes via hydrophobic fusion peptides, and then pull cell and virus membranes into proximity, through a refolding process that ends in membrane fusion and stable “postfusion” helical bundles (13, 14). Pre- and postfusion S protein structures (15, 16), structural intermediates on the refolding pathway (13, 17), and adaptive variations impacting the refolding process (18–20) indicate that these cell entry dynamics are under powerful selective forces, potentially influencing CoV transmissibility.

Numerous substitutions and deletions have been identified in the S proteins of viruses associated with the COVID19 pandemic (21, 22). Our broad aims were to identify those consequential changes that alter the dynamics of virus-cell entry and infection. To this end, we focused on comparing SARS-CoV-2 S with the S proteins of related sarbecoviruses. There are notable distinctions. First, SARS-CoV-2 S proteins have S1 amino-terminal domain (NTD) loops that are divergent both in sequence and in length from those of other sarbecoviruses (23; see also Fig. S1 in the supplemental material). Second, SARS-CoV-2 proteins have structurally divergent S1 receptor-binding domains (RBDs) that bind with relatively high affinity to human angiotensin-converting enzyme 2 (hACE2) (23, 24). Third, unlike several related sarbecoviruses, SARS-CoV-2 S proteins contain a substrate site for cleavage by the cellular protease furin, which upon proteolysis divides S protomers into noncovalently associated S1 and S2 portions (25, 26).

10.1128/mBio.01590-21.1 FIG S1 CoV spike protein indel hotspots. Sequence alignments for CoV S protein NTDs and CTDs of selected embecoviruses (a), sarbecoviruses (b), merbecoviruses (c), and nobecoviruses (d). Residue percent identity is colored in shades of blue. Indel hotspots are indicated by red boxes. The following sequences were used: MHV-JHM (GenBank accession no. YP_209233.1), beta-CoV (GenBank accession no. AAA66399.1), rodent CoV (GenBank accession no. ATP66744.1), OC43 (GenBank accession no. QEG03814.1), HKU24 (GenBank accession no. YP_009113025.1), HKU1 (GenBank accession no. ADN03339.1), SARS-CoV-2 (GenBank accession no. QPI75814.1), SARS-CoV (GenBank accession no. AAP13441.1), bat SARS-related (SARSr)-CoV RaTG13 (GenBank accession no. QHR63300.2), bat SARSr-CoV WIV1 (GenBank accession no. AGZ48828.1), bat SARSr-CoV Rp3/2004 (GenBank accession no. AAZ67052.1), bat SARSr-CoV Rc-o319 (GenBank accession no. BCG66627.1), MERS-CoV (GenBank accession no. QFQ59587.1), bat MERSr-CoV NL140422 (GenBank accession no. AVV62537.1), bat MERSr-CoV Vs-CoV-1 (GenBank accession no. BBJ36008.1), bat MERSr-CoV 206645-63/2011 (GenBank accession no. AUM60024.1), HKU31 (GenBank accession no. QGA70692.1), BtVs-BetaCoV/SC2013 (GenBank accession no. AHY61337.1), rousettus bat CoV (GenBank accession no. AOG30822.1), BtRt–beta-CoV/GX2018 (GenBank accession no. QDF43840.1), HKU9 (GenBank accession no. YP_001039971.1), and MCL_19_Bat_606_10 (GenBank accession no. QJX58383.1). NTD indels characterized in this study are labeled (panel b, loops 1, 2, and 3). Download FIG S1, EPS file, 2.6 MB. Copyright © 2021 Qing et al. 2021 Qing et al. https://creativecommons.org/licenses/by/4.0/ This content is distributed under the terms of the Creative Commons Attribution 4.0 International license.

In strong support of the biological significance of these three distinctive features, recent highly transmissible variants of concern harbor deletion and substitution mutations in all three regions (5). Of these three regions, two are partially understood in mechanistic detail: the affinity of the RBD-hACE2 interactions (27, 28) and the proteolytic cleavage into S1 and S2 fragments (25, 26), both of which are considered relevant to SARS-CoV-2 human emergence and transmission. This leaves the operating mechanisms of the divergent NTD loops open to further investigation. The NTD loops may be part of a structure that binds viruses to attachment factors, of which there are several candidates (29–33), each potentially imposing selective forces. The NTD loops are recognized by virus-neutralizing antibodies (34, 35), making it possible that immune pressures drive their divergence into alternative structures. Yet another possibility is that the NTDs operate to control S protein unfolding transitions required for virus-cell entry (36). Here, we explored the last possibility. We generated results implicating SARS-CoV-2 NTDs in the cell binding and membrane fusion stages of virus entry.

## RESULTS

### Hypervariability in coronavirus spike NTDs.

CoV S proteins are among the most variable coronavirus-encoded proteins. Of note, S protein variability includes both indels and substitutions. Indels, generated by discontinuous CoV transcriptional processes (37), are concentrated in S protein NTDs and RBDs, and they stand out as key features of variability in all four betacoronavirus groups (see Fig. S1 in the supplemental material). Several decades ago, the biological significance of vestigial RBD indels was investigated in some of the embecoviruses, where specific indels were correlated with S protein stability and reduced spike-directed membrane fusion catalysis (38, 39). However, the effects that NTD indels may have on S protein structural dynamics and catalytic function remain unclear.

**FIG 1:**
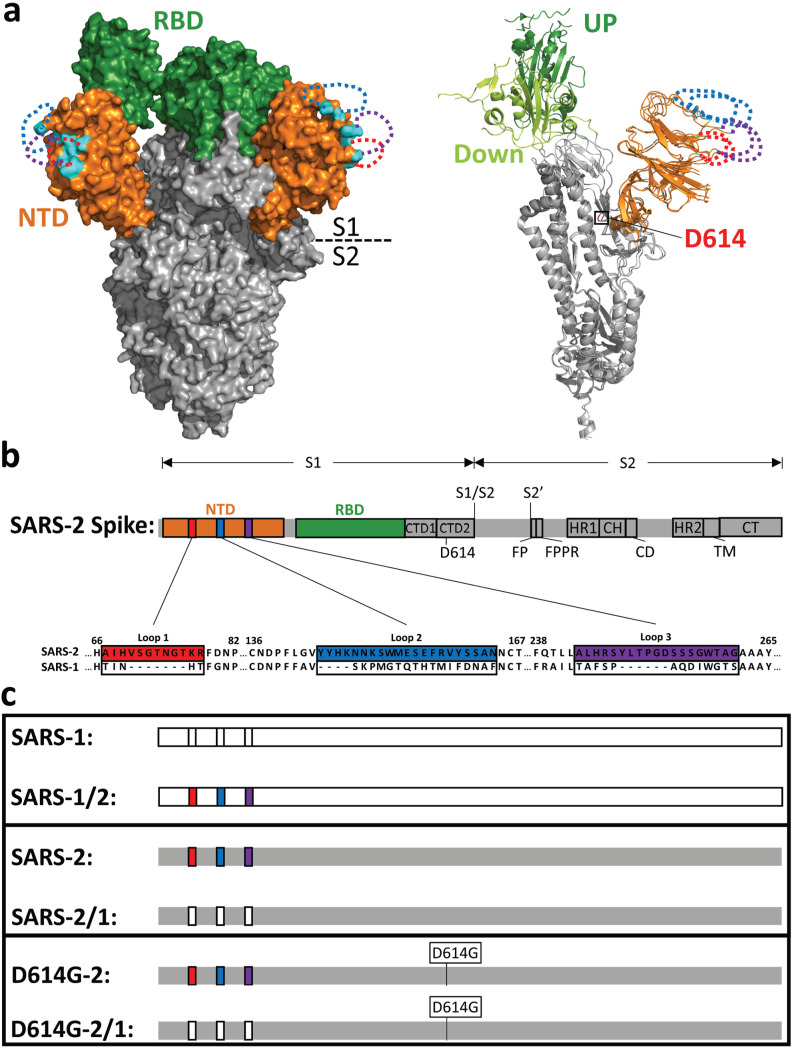
NTD loops on the SARS-CoV-2 spike protein. (a, left) SARS-CoV-2 S cryo-EM structure (PDB accession no. 6VSB) in surface representation. The N-terminal domain (NTD; orange) and receptor-binding domain (RBD; green) are depicted in the context of the trimer ectodomain (gray). Unresolved NTD loops are depicted by dotted lines (red, blue, and purple). Resolved residues nearest to the NTD loops are in cyan. (Right) A single S monomer is depicted in a ribbon diagram with RBD-up (dark green) superimposed on RBD-down (light green). D614 is labeled in red. (b) Linear depiction of the complete SARS-CoV-2 S protein. The NTD (orange), the RBD (green), C-terminal domain 1 (CTD1), C-terminal domain 2 (CTD2), D614, S1/S2 priming, the S2′-activating cleavage sites, the fusion peptide (FP), the fusion peptide-proximal region (FPPR), heptad repeat 1 (HR1), the central helix (CH), the connector domain (CD), heptad repeat 2 (HR2), the transmembrane span (TM), and the cytoplasmic tail (CT) are depicted. Three NTD loops are highlighted in colored boxes (red, blue, and purple) and enlarged to reveal the amino acid sequences of SARS-CoV-2-S (GenBank accession no. NC_045512.2) and corresponding SARS-CoV-S (GenBank accession no. AY278741.1), which were exchanged. (c) Recombinant spike protein pairs. (Top) SARS-1 in comparison with SARS1/2 (SARS-2 NTD loops in color); (middle) SARS-2 in comparison with SARS-2/1 (SARS-1 NTD loops not in color); (bottom) SARS-2 and SARS-2/1 in the D614G background.

Both NTD and RBD indels distinguish several sarbecovirus S proteins (22) (Fig. S1). In a comparison of SARS-CoV-1 and SARS-CoV-2, NTD variations are evident at three locations, highlighted in red, blue, and purple in Fig. 1a and b. Both substitutions and indels are evident. Perhaps expectedly, the sites of substitution and indel hypervariability are poorly resolved in structures of stabilized SARS-CoV-2 ectodomains (13, 23, 24). The variable regions are therefore sketched as dotted lines in Fig. 1a, with each dotted line forming a loop. The three loops together form a prominence at the distal part of each NTD (cyan and dotted loops in Fig. 1a). Of note, this region is part of a SARS-CoV-2 antigenic supersite (40, 41).

Guided by the SARS S structures and the primary sequence variabilities, we exchanged nucleotide sequences encoding these hypervariable portions of the NTDs. In this way, we expected to preserve the SARS-1 and SARS-2 NTD core structures while varying distal loop length and composition. Specifically, the smaller SARS-1 regions from residues 71 to 75 (loop 1), 141 to 157 (loop 2), and 236 to 248 (loop 3) were reciprocally exchanged with larger SARS-2 regions from residues 67 to 78 (loop 1), 144 to 164 (loop 2), and 243 to 261 (loop 3). The exchanged residues did not include any cysteines and did not generate alternative *N*-glycosylation patterns in the recombinant proteins.

We compared the S proteins as isogenic pairs, as illustrated in Fig. 1c. SARS-1 S was compared to SARS-1/2 (SARS-1 with the larger SARS-2 loops). Conversely, SARS-2 S was compared with SARS-2/1 (SARS-2 with the smaller SARS-1 loops). We also introduced a SARS-2 S-stabilizing D614G change (4) into SARS-2 and SARS-2/1 (Fig. 1a; protomer alpha carbon tracing). These constructs were designated D614G-2 and D614G-2/1, respectively (Fig. 1c). Inclusion of the D614G substitution allowed us to consider potential epistatic NTD-D614G control mechanisms.

### Hypervariable NTD loops control S protein stability and membrane fusion potential.

**FIG 2:**
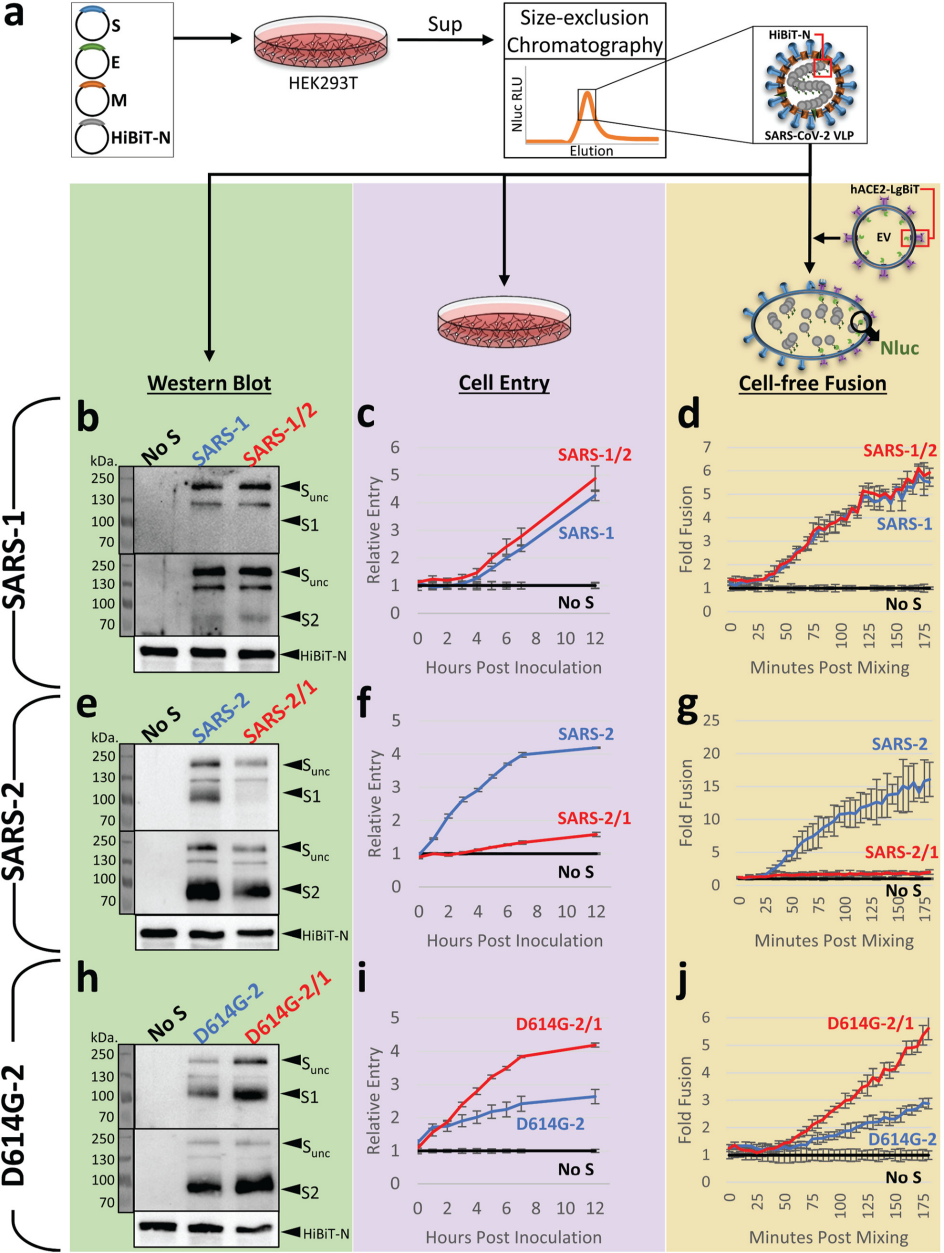
Hypervariable NTD loops control S protein stability and membrane fusion potential. (a) Plasmids encoding SARS-CoV-2 spike (S), envelope (E), membrane (M), and amino-terminal HiBiT-tagged nucleoprotein (HiBiT-N) were expressed in HEK293T cells, supernatants (sup) were harvested after 2 days, and HiBiT-VLPs were purified by size exclusion chromatography. VLPs were compared as isogenic pairs: SARS-1 versus SARS-1/2 (panels b to d), SARS-2 versus SARS-2/1 (panels e to g), and D614G-2 versus D614G-2/1 (panels h to j). Each pair was evaluated by Western blot (left), cell entry (middle), and cell-free fusion (right) assays. Western blot assays detected uncleaved S (S-unc), S1, S2, and HiBiT-N. Cell entry assays detected HiBiT-VLP entry into ACE2-LgBiT/hTMPRSS2 target cells. The cell entry data are presented relative to the cell entry of control inoculations of spikeless (No S) VLPs. Cell-free fusion data are presented as HiBiT-VLP:ACE2-LgBiT EV fusion levels relative to data under control conditions with spikeless VLPs. For the cell entry and cell-free fusion data, the error bars present standard deviations (SD) from three technical replicates (*n *= 3), with data being representative of three biological repeats.

We utilized a coronavirus-like particle (VLP) platform to determine whether the reciprocal exchanges of NTD loop regions affect changes to virus infection stages. VLPs allowed us to track S proteins through the stages of virus particle assembly, secretion from producer cells, extracellular particle stability, and subsequent particle entry into target cells through virus-cell membrane fusion. To produce VLPs incorporating the various SARS-1 and SARS-2 S variants, each S gene construct was combined with the SARS-CoV-2 E (envelope), M (membrane), and N (nucleocapsid) genes, and the four were then cotransfected into HEK293T cells. The N genes were engineered to include a nanoluciferase (Nluc) “HiBiT” fragment (42, 43), making it so that transfected cells produced and secreted HiBiT-N-containing VLPs. Secreted VLPs were harvested and purified using size exclusion chromatography (Fig. 2a). Notably, the VLPs were readily detectable throughout these particle purification procedures by complementing the internal HiBiT tags with Nluc “LgBiT,” which forms an easily quantified Nluc enzyme activity (Fig. 2a and references 42 and 43).

Western immunoblotting demonstrated that all VLPs contained virion S, E, M, and N proteins, supporting the contention that VLPs are faithful reflections of authentic SARS-CoV-2 virions (43). Here, we focused further on VLP S proteins, as well as S proteolytic cleavage products S1 and S2, so that S1-S2 heterodimer stabilities might be assessed. We found that the NTD exchange had relatively little effect on N-to-S ratios in the context of SARS-1 (Fig. 2b). SARS-1 S proteins remain largely uncleaved throughout particle genesis and secretion (44), and therefore, S1 fragments were not abundant on the purified VLPs (Fig. 2b). However, SARS-2 S proteins are subject to furin-mediated cleavage (23), making for prominent VLP-associated S1 and S2 (Fig. 2e). For SARS-2, the NTD exchange in SARS-2/1 effected a profound S protein alteration that was most evident by the nearly complete absence of S1 on VLPs (Fig. 2e). This instability was not observed with VLPs harboring the D614G substitution (Fig. 2h). In fact, in relation to the N proteins that serve as internal standards, the D614G-2/1 VLPs had more intact S1-S2 heterodimers than any of the variants analyzed in this study (Fig. 2h). These findings demonstrate that the sequence and length of hypervariable NTD loops control S protein heterodimer stabilities.

The HiBiT-containing VLPs were used to evaluate CoV cell entry in biosafety level 1 (BSL-1) assays that specifically isolate the initial cell entry stage of infection. First, we constructed clones encoding hACE2 with cytoplasmic (carboxy-terminal) LgBiT and then introduced hACE2-LgBiT and hTMPRSS2 cDNAs into HeLa cells to generate virus susceptibility (45). Next, we inoculated the cells with HiBiT VLPs and quantified HiBiT-LgBiT complementation (Nluc accumulation) over time. Using the normalized Nluc levels as a measure of successful VLP cell entry, we noted that NTD modifications in the SARS-1 background had no effect on VLP cell entry (Fig. 2c). In contrast, the NTD modifications in the SARS-2 background had profound effects. Relative to parental SARS-2, NTD-altered SARS-2/1 had ∼10-fold less entry (Fig. 2f). This finding was consistent with the loss of S1 from the NTD-altered VLPs (Fig. 2e). The D614G substitution had a notable restorative effect. Relative to parental D614G-2, the NTD-altered D614G-2/1 had ∼3-fold more entry (Fig. 2i), consistent with S1 retention on the NTD-altered VLPs (Fig. 2h). These findings ascribe both loss- and gain-of-function properties to changes in the distal NTD loops of SARS-CoV-2.

VLP cell entry required hACE2 and hTMPRSS2 on target cells. It is known that hACE2 incorporates into extracellular vesicles (EVs) and secretes from cells (46). We found that EVs with incorporated ACE2-LgBiT were readily harvested and purified from target cell supernatants (Fig. S2a and b). This prompted us to consider whether ACE2-containing EVs might substitute for target cells, making for cell-free assays of VLP fusion with EV target membranes. We incubated purified ACE2-LgBiT EVs with HiBiT VLPs, using trypsin in place of hTMPRSS2 to cleave/activate S protein fusion catalysis (47), and then measured fusion-dependent complementation of HiBiT and LgBiT into Nluc. This cell-free VLP-EV fusion assay was highly sensitive, revealing VLP fusion signals nearly 3 log_10_ over background, with the signals being linearly proportional to VLP concentration over a 2.5-log_10_ range (Fig. S2c and d). Using this cell-free assay format, pairwise comparisons of VLPs were made, as schematized in Fig. 1c. SARS-1 and SARS-1/2 VLP fusions were indistinguishable (Fig. 2d), SARS-2 VLP fusion exceeded that of SARS-2/1 by ∼10-fold (Fig. 2g), and D614G-2 fusion trailed that of D614G-2/1 by ∼3-fold (Fig. 2j). These findings accorded with TMPRSS2-activated cell entry data (Fig. 2c, f, and i). Together, the results in Fig. 2 demonstrate that NTD loops control S1-S2 heteromeric stability and membrane fusion potential.

10.1128/mBio.01590-21.2 FIG S2 Characterization of cell-free fusion. (a) Detection of hACE2-LgBiT via postlysis LgBiT-HiBiT complementation by addition of passive lysis buffer (Promega), the Nluc substrate (Promega), and the HiBiT lysate. Fractions with high RLU (fractions 7 and 8) correlated with the presence of hACE2-LgBiT EVs. (b) Western blot detection of proteins from fractions 7 and 8 from panel a. hACE2-LgBiT was detected by both hACE2 antibody and the HiBiT lysate. (c) Serial dilutions of SARS-2 VLPs were mixed with hACE2-LgBiT EVs, the Nluc substrate, and trypsin (10 ng/μl) at 4°C. Nanoluciferase (Nluc) was then measured every 5 min for 180 min following a shift to 37°C. Error bars present standard deviations (SD) from three technical replicates (*n *= 3). (d) One hundred eighty-minute RLU values from panel c were extrapolated and plotted against the VLP input. Data are representative of two biological repeats. Download FIG S2, EPS file, 2.0 MB. Copyright © 2021 Qing et al. 2021 Qing et al. https://creativecommons.org/licenses/by/4.0/ This content is distributed under the terms of the Creative Commons Attribution 4.0 International license.

### Hypervariable NTD loops control RBD exposure.

**FIG 3:**
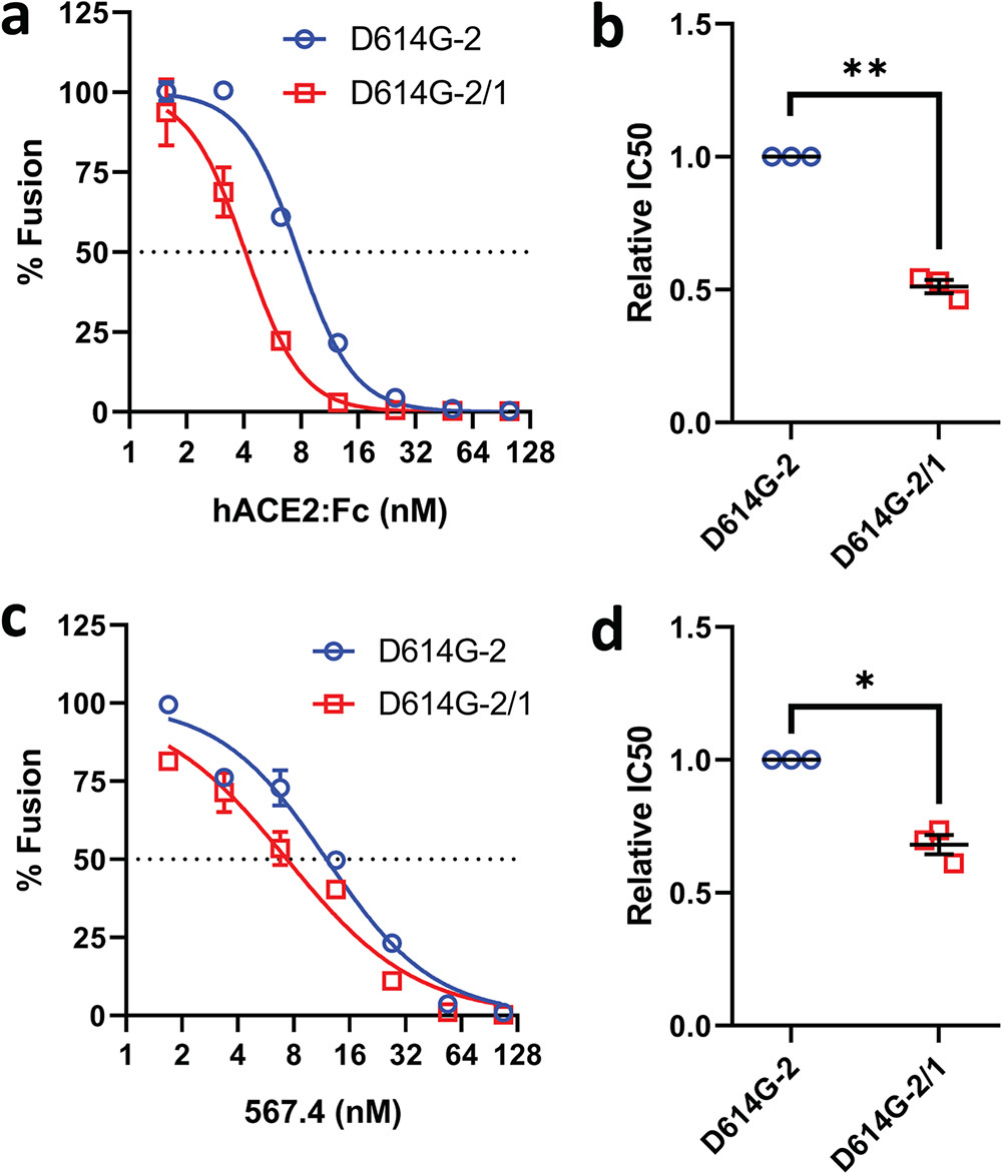
Hypervariable NTD loops control RBD exposure. (a and c) Serial dilutions of hACE2-Fc (a) or RBD MAb 567.4 (c) were incubated with D614G-2 or D614G-2/1 VLPs for 30 min at 37°C, and cell-free VLP-EV fusions were then measured. Data trendlines were normalized to vehicle fusion levels. Error bars present SD. (b and d) Experiments were repeated three times, and IC_50_ values were calculated from the three fitted normalized response trendlines. Relative IC_50_ data are presented for hACE2-Fc (c) and RBD MAb 567.4 (d). Statistical analyses were assessed by an unpaired Student *t* test (*, *P < *0.05; **, *P* < 0.01).

The complete separation of S1 from virus particles is a near-end-stage event in the transition from pre- to postfusion S protein conformations, coming only after more subtle NTD and RBD rearrangements (13, 17, 48). We aimed to further understand how NTD loops influence the conformational changes that precede S1 separation. Among these rearrangements are the dynamic RBD elevations that position the S proteins for hACE2 receptor interactions (47, 49). We compared the RBD dynamics on D614G-2 and D614G-2/1 VLPs, because these two variants have similar S1-S2 densities yet differ in NTD loops and ACE2-dependent cell entry activities. To compare RBD positioning, we introduced soluble hACE2-Fc receptors into cell-free fusion assays (Fig. 2j) and assessed soluble receptor interference. Interference with D614G-2/1 (50% inhibitory concentration [IC_50_] = 5 nM) was 2-fold greater than with D614G-2 (Fig. 3a and b). As the two VLPs have identical RBDs, this finding argues that changes in the hypervariable NTD loops can control RBD repositioning into the standing states that bind ACE2. Recurrent display of elevated RBDs to target cell ACE2 may explain the gain of D614G-2/1 VLP cell entry function.

Similar cell-free fusion assays were performed in the presence of an RBD-specific monoclonal antibody (MAb). Relative to D614G-2 VLPs, the recombinant D614G-2/1 VLPs were moderately more sensitive to neutralization (Fig. 3c and d). These findings further support the contention that hypervariable NTD loops control RBD exposures.

### Hypervariable NTD loops and protease exposure.

**FIG 4:**
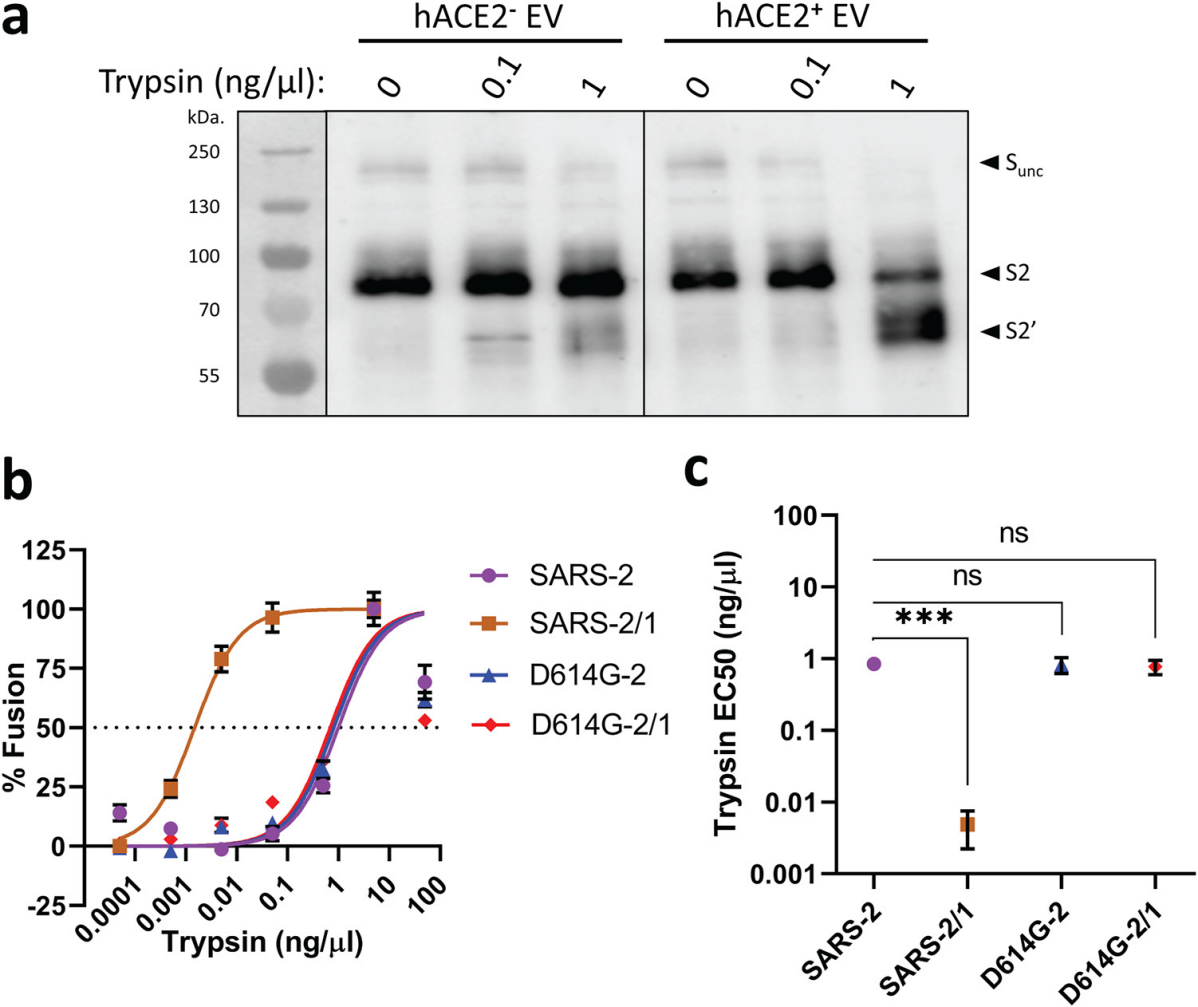
NTD loops control protease-triggered membrane fusion. (a) D614G-2 VLPs were incubated with hACE2-negative or hACE2-positive EVs for 30 min at 37°C in the presence of the indicated trypsin concentrations, and trypsin cleavage products were identified by Western blotting. Uncleaved S (S-unc), S2, and S2′ cleavage products are indicated. (b) The indicated SARS-CoV-2 VLPs were evaluated in cell-free VLP-EV fusion assays at the indicated trypsin concentrations. Fusion readouts were taken after 3 h at 37°C, and plotted data trendlines were normalized to the highest measured fusion levels. Error bars present standard errors (SE) of the means. (c) Cell-free VLP-EV fusion assays were repeated three times and trypsin EC_50_ values calculated from the fitted normalized response trendlines. Statistical analyses were assessed by an unpaired Student *t* test (***, *P < *0.001; ns, not significant).

During transitions toward membrane fusion, S proteins are cleaved by host proteases at a site that is largely buried in the prefusion state (23, 50). Cleavage at this “activating” S2′ site is required to unleash S proteins for subsequent membrane fusion-catalyzing rearrangements. We aimed to determine whether NTD loops influence S2′ substrate site exposure. We compared two VLP pairs for S2′ cleavage-dependent fusion activation: SARS-2 with SARS-2/1 and D614G-2 with D614G-2/1. In cell-free fusion assays, trypsin cleaved ACE2-associated VLP S proteins at two positions, one consistent with scission at the activating S2′ site (Fig. 4a). To identify VLP sensitivity to this S2′ cleavage, trypsin was titrated over a 7-log_10_ range and the cell-free fusions resulting from the trypsin cleavages were then measured. The SARS-2/1 VLPs were convincingly hypersensitive to trypsin-mediated fusion activation (Fig. 4b). From the titration data, fusion-activating trypsin concentrations were defined in terms of the 50% effective concentration (EC_50_) (Fig. 4c), revealing that the highly labile SARS-2/1 VLPs with unstable S1-S2 heterodimers were ∼100 times more sensitive to proteolytic activation than the other three more stable VLPs. These findings suggest that selective forces driving S protein stability oppose those forces driving facile proteolytic activation of membrane fusion.

### S protein domain exposures and virus vulnerability.

**FIG 5:**
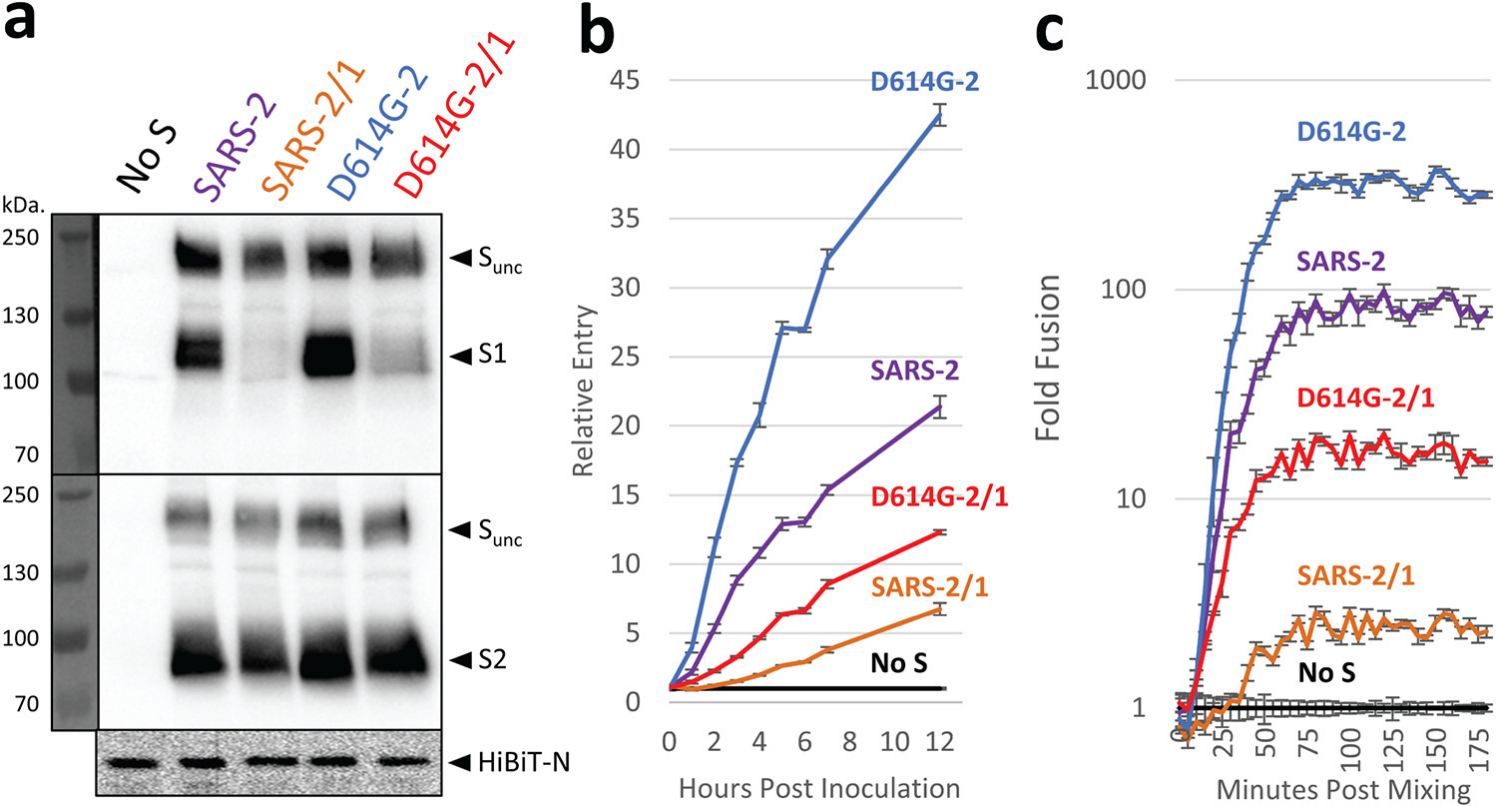
NTD loops control SARS-2 spike stability. (a) VLPs were pelleted through 20% sucrose, resuspended, and evaluated by Western blotting. Western blot assays detected uncleaved S (S-unc), S1, S2, and HiBiT-N. (b) The same resuspended VLPs were evaluated in cell entry assays. Time course cell entry data are presented relative to those of control inoculations of spikeless (No S) VLPs. (c) The same resuspended VLPs were evaluated in cell-free VLP-EV fusion assays. Time course fusion data are presented as fold changes from the no-S condition containing “spikeless” VLPs. (b and c) Error bars present standard deviations (SD) from three technical replicates (*n *= 3). Data shown are representative of three biological repeats.

A propensity for RBD and protease substrate exposure can facilitate virus-cell entry but may leave extracellular viruses vulnerable to inactivation. We were surprised that D614G-2/1 VLPs appeared as exceptions to this pattern, as these particles exhibited more RBD exposure and more cell entry than parental D614G-2, all without compromising VLP stability. We further evaluated the maintenance of D614G-2/1 VLPs and found that they were indeed identical to parental D614G-2 in thermal (37°C) stability over a 40-h time period (Fig. S3). However, the D614G-2/1 particles were notably fragile under certain experimental conditions. Upon centrifugation through sucrose cushions, D614G-2/1 VLPs lost S1 (Fig. 5a) and in turn had diminished capacity for cell entry (Fig. 5b) and cell-free membrane fusion (Fig. 5c). Thus, the pattern holds; selective pressures for effective cell entry oppose those instilling S protein stability.

10.1128/mBio.01590-21.3 FIG S3 NTD loops control SARS-2 spike stability. (a) VLPs bearing the indicated spike proteins were incubated for 0, 24, or 41 h at 37°C before they were mixed with hACE2-LgBiT EVs, the substrate, and trypsin for cell-free fusion. Data trendlines were normalized to 0-h fusion levels. Error bars represent standard deviations. (b) The experiment in panel a was repeated three times. Half-life (*t*_1/2_) values were calculated from the three fitted normalized response trendlines. Statistical analyses were assessed by an unpaired Student *t* test. Color schemes are the same as in panel a. Download FIG S3, EPS file, 0.6 MB. Copyright © 2021 Qing et al. 2021 Qing et al. https://creativecommons.org/licenses/by/4.0/ This content is distributed under the terms of the Creative Commons Attribution 4.0 International license.

### Hypervariable NTD loops and virus attachment to target cells.

**FIG 6:**
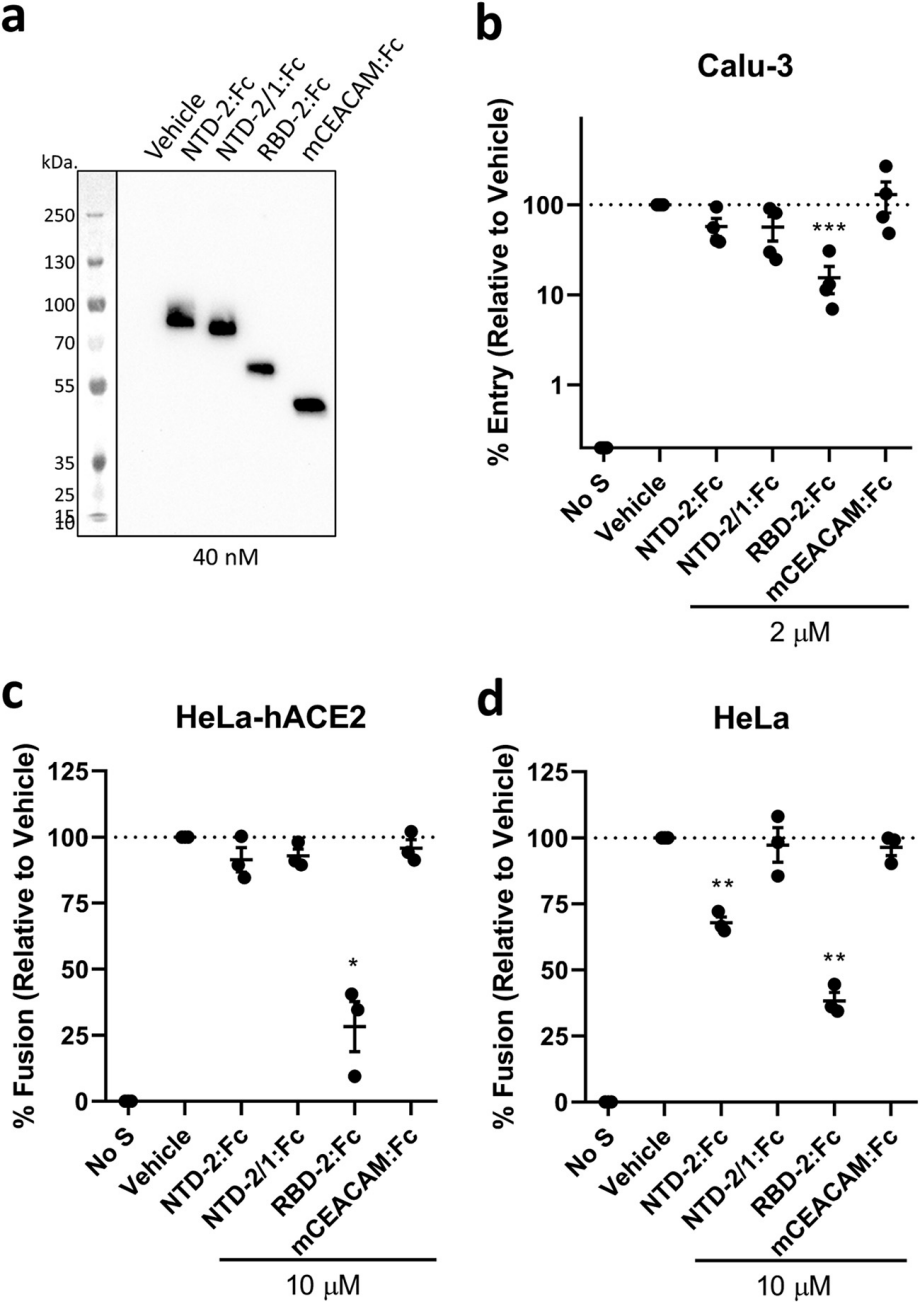
NTDs require loop structures to interfere with S-directed transduction and cell-cell fusion. (a) Western blot analysis of purified NTD, RBD, and CEACAM:Fc proteins (20 μl/lane at 40 nM). (b) VSV-Fluc PPs bearing SARS-CoV-2 S were inoculated onto Calu-3 cells together with the vehicle or the indicated Fc constructs (2 μM). After 18 h, Fluc levels were measured to reflect PP cell entry and are presented as percent entry relative to that during vehicle control conditions. Each data point represents averages (*n *= 4 replicates) from independent experiments (*n* = 4 experiments). Error bars present standard errors (SE) of the means. Statistically significant deviations from vehicle control values were assessed by an unpaired Student *t* test (***, *P < *0.001). (c and d) Cell-cell fusion assays were established with HeLa-hACE2 (c) or HeLa (d) target cells in the presence of the indicated Fc constructs (10 μM). Rluc levels were measured to reflect cell-cell fusion and are presented as percent cell fusion relative to that under vehicle control conditions. Each data point represents averages (*n *= 4 replicates) of Rluc levels measured at 4, 12, and 22 h after cocultivation. Error bars present standard errors (SE) of the means. Statistically significant deviations from vehicle control data were assessed by an unpaired Student *t* test (*, *P < *0.05; **, *P < *0.01). Data are representative of two biological repeats.

While the collective results indicated that NTD loops control S protein stability, virus-cell entry, and virus-cell membrane fusion, they did not discount additional roles in direct virus-cell attachment. It remained possible that the high cell entry potential of D614G-2/1 VLPs (Fig. 2i) came from SARS-2/1 exchanges that increased direct NTD-cell binding. CoV NTDs do indeed attach to cell surface carbohydrates (51, 52) or proteins (53, 54), and the SARS-CoV-2 NTDs in particular are proposed ligands for carbohydrates (31, 32) and proteins (29, 30). To determine whether NTDs operate in virus-cell binding, SARS-CoV-2 NTD-2:Fc and RBD-2:Fc proteins (Fig. 6a) were introduced during SARS-CoV-2 pseudoparticle (PP) transductions. Transductions into human airway-derived Calu-3 cells, which are hACE2 positive and highly susceptible to SARS-CoV-2 (25, 45), were reduced significantly by RBD-2:Fc, but not by NTD-2:Fc (Fig. 6b). Similar findings came from evaluation of HeLa-hACE2 cells, where RBD-2:Fc but not NTD-2:Fc interfered with S protein-mediated fusion into syncytia (Fig. 6c). While these results argued against a role for NTDs in virus-cell entry, it was possible that RBD:hACE2 interactions dominated the S-mediated entry and cell fusion processes, leaving no observable role for NTDs. HeLa cells express endogenous hACE2 at low levels, below that required for SARS-CoV-2 infection (1) but not so low as to preclude S protein-mediated fusion of the cells into syncytia (42). With HeLa cells, NTD-2:Fc reduced S-mediated cell fusions by about 30%, while NTD-2/1:Fc, which displays altered SARS-1 NTD loops, effected no change in fusions (Fig. 6d). While these findings indicate that the larger NTD-2 domains contribute moderately to S protein binding to HeLa cells, they do not support suggestions that direct binding of the smaller NTD-2/1 accounts for the enhanced entry and fusion potential of the D614G-2/1 variant. We conclude that the NTD variations evaluated in this study increase virus entry by mechanisms unrelated to direct NTD attachment to cells.

## DISCUSSION

Metastable viruses are durably enclosed extracellular particles yet poised for opening at cell entry. The CoV S proteins directing this opening process must maintain both stable extracellular native (prefusion) states and dynamic responses to target cell receptors and proteases during virus-cell entry. Harsh extracellular environments select against S protein mobilities that can spontaneously inactivate viruses, while facilitated cell entry selects for S protein flexibilities that reveal receptor-binding and membrane fusion domains. These opposing selective pressures drive adaptive variations that rebalance metastable “set points.” For example, murine CoVs acquire S protein deletions during cell culture passaging, acquiring extracellular stability with concomitant reductions in membrane fusion triggering (38, 39, 55, 56). Adaptive changes in Middle East respiratory syndrome CoV (MERS-CoV) also change S-mediated fusion thresholds (57, 58). More recent studies of SARS-CoV-2 revealed cell culture adaptations that include deletions of furin protease substrates, with resultant uncleaved S proteins stabilized against S1 shedding but concomitantly less susceptible to TMPRSS2 cleavage activation of membrane fusion (26, 59, 60). The now-prevalent D614G change in SARS-CoV-2 also stabilizes S proteins noncovalently, promoting S1 retention (4, 36) and extracellular infectivity (20, 61), but potentially with reduced susceptibility to activating serine proteases (62).

This report identifies another locus of S protein variation that controls CoV metastability. The control elements were in hypervariable SARS-CoV-2 NTD loops, an unanticipated location that is distant from receptor-binding and activating protease substrate sites and also far from S protein interdomain connections. In speculating on how NTD loops operate indirectly to control S protein stability, we noted that the loops are not visible in cryoelectron microscopy (cryo-EM) images of unliganded S proteins (17, 24) but are resolved when fixed into position by bound neutralizing antibodies (35). One conjecture is that restriction of the normally dynamic loops by antibodies, or by deletion of flexible glycine-rich stretches (Fig. 1), rigidifies the NTDs more globally or changes the pitch of NTDs relative to the 3-fold S trimer axes, which in turn distorts the interdomain contacts regulating S1-S2 separation and resultant virus fusion. SARS-CoV-2 NTDs do indeed contact “630 loops” that control interdomain connections at critical S1-S2 interfaces (36). The hypervariable NTD regions regulated these interdomain contacts, in that they impacted both hACE2:Fc binding and S1-S2 separation, which we interpret as NTD control of the more C-terminal RBDs into ACE2-accessible “up” orientations (47). With RBDs “up” and then maintained in this position by bound ACE2, interdomain contacts further rearrange to permit membrane fusion and near-end-stage S1 shedding (49, 63). Our results appear consistent with NTDs and RBDs operating cooperatively to reveal receptor-binding and membrane fusion-catalyzing elements in the S proteins. Future atomic-resolution imaging will be necessary to shed more light on cooperative NTD and RBD rearrangements.

The impact of NTD hypervariability depends on the S protein background. For the uncleaved SARS-CoV (“SARS-1”) S proteins, NTD loop exchanges did not effect measurable changes in VLP entry and membrane fusion. We suggest that the stability of SARS-1 S is controlled by covalent S1-S2 linkage and other interfaces that are not influenced by distal NTD loops. For cleaved SARS-CoV-2 (“SARS-2”) S proteins, the NTD loop exchanges were profoundly destabilizing, but the D614G substitution that is known to connect S1 and S2 (4) and enhance virus transmissibility (3, 64, 65) restored the prefusion S1-S2 structure. S structures show that the D614G change allows packing of “630 loops” between NTD and CTD1 domains (36), and functional data correlate this interdomain packing with S1-S2 stability, even when one S protomer has an RBD in a precarious “up” position (66). Although NTD loop-altered SARS-2 S structures are not yet available, we speculate that the NTD loops further adjust these D614G-630 loop control elements in ways that generate gain-of-function S proteins with enhanced hACE2 binding and membrane fusion characteristics. Taken in light of SARS-CoV-2 evolution through 2020, we further speculate that the initial springtime 2020 expansion of D614G variant SARS-CoV-2 generated overstabilized viruses that could accommodate adaptive destabilizing changes in NTDs and possibly other S domains. In fact, NTD indels in SARS-CoV-2 variants of concern were observed only after the stabilizing D614G substitution (67). That a genetic drift around metastable set points can potentially generate hyper-fusogenic CoVs with enhanced cell entry potential is an important consideration in understanding CoV cell entry, transmission, and pathogenicity.

This report also highlights relationships between enhanced cell entry potential and virus stability. The D614G VLPs harboring the smaller NTD loops showed enhanced hACE2 interactions and increased cell entry, yet they were unstable and shed S1 during a relatively gentle VLP purification process (Fig. 5). Similarly, the SARS-2 VLPs with the smaller NTD loops showed dramatically higher sensitivity to fusion-activating trypsin protease (Fig. 4), making them potentially capable of broad cell entry, yet they were so unstable that their entry potential decayed rapidly in cell culture media. These findings highlight the opposing selective pressures that bear on CoVs as they adapt in nature.

NTD hypervariability may also be driven by selective binding to host cell attachment factors. Indeed, our original hypothesis was that the hypervariable NTD loops are part of a cell-binding motif, given their distal location on the S protein trimers (Fig. 1) and given that the NTDs of other beta-CoVs are established cell receptor-binding domains (52, 54). Exploring this hypothesis, we obtained evidence that the NTDs bind to cell surfaces (Fig. 6). DC-SIGN may be an NTD attachment factor, as it binds SARS-CoV (68) and was recently shown to interact with SARS-CoV-2 at glycan N149 (29), a residue within NTD loop 2. Very low levels of HeLa cell surface DC-SIGN (69) might explain the modest NTD binding. Alternatively, sialic acids may be virus-binding ligands, as they have been recently proposed to bind at or near residues comprising NTD loop 1 (32). Other putative NTD-interacting factors (30, 31) are possible host factors, as are NTD-directed allosteric effects that influence cell attachment through other S protein domains (70). While we did not find evidence favoring direct NTD cell binding as a step toward enhanced entry (Fig. 6), we remain open to NTD-facilitated virus entry in other contexts. Other infection contexts may even reveal that NTD cell binding reorients RBDs and fusion domains for effective entry.

The COVID-19 pandemic is now dominated by several variants of concern (VOCs) (5). Among these VOCs, several have NTD deletions; for example, the prevalent B.1.1.7 variant has deletions of residues 69 to 70 and 144 to 145 within NTD loops 1 and 2, respectively (71) (Fig. 1 and see Fig. S4 in the supplemental material). Furthermore, viral RNAs have been isolated from persistently infected patients, with sequencing revealing in-frame recurrent deletion regions (RDRs) at or near the same three NTD loops that were evaluated in our study (22) (Fig. S4). In correlating the results of our study with these recurrent NTD deletions, we must note our limitations. First, our study does not investigate NTD changes in the context of authentic, replication-competent SARS-CoV-2. However, we emphasize that the VLPs employed in this study faithfully reflect authentic SARS-CoV-2 entry far more closely than that of frequently employed pseudo-SARS-2 viruses (42, 43) (Fig. S5). The cell-free VLP fusion assays utilized in this study also have broad utility, in that CoV entry can be analyzed quantitatively in carefully controlled *in vitro* settings. Furthermore, as noninfectious BSL-1 surrogates of SARS-CoV-2, the VLPs are the appropriate reagents for evaluating variants that might present significant biohazards if constructed into replication-competent viruses. Second, the deletions that we constructed by SARS-CoV/SARS-CoV-2 exchanges do not have precisely the same breakpoints and lengths as the naturally occurring VOC and RDR deletions, and in these VOCs, several other S protein substitutions may operate in metastable control. Experiments with natural VOC VLPs are in progress. That stated, the findings in our study indicate that NTD deletions should be investigated in ways that go beyond their known contributions to antigenic variability (22, 72) with an additional distinct focus on their potential for establishing alternative metastable states that increase virus transmissibility.

10.1128/mBio.01590-21.4 FIG S4 Naturally occurring SARS-CoV-2 variants contain deletions at or near the NTD loops. NTD sequence alignments are shown with residue identities colored in shades of blue. Sequences of the three NTD loops are within the red boxes. Sequences shown include SARS-CoV-2 (GenBank accession no. QPI75814.1), B.1.1.7 (GenBank accession no. QTC11018.1), B.1.351 (GenBank accession no. QTE05846.1), P.1 (GenBank accession no. QVQ47339.1), B.1.617.2 (GenBank accession no. QVI56963.1), four variants identified from patients persistently infected with SARS-CoV-2 (GISAID accession no. EPI_ISL_582112, EPI_ISL_476148, EPI_ISL_581793, and EPI_ISL_583430), and SARS-CoV (GenBank accession no. AAP13441.1). Download FIG S4, EPS file, 2.1 MB. Copyright © 2021 Qing et al. 2021 Qing et al. https://creativecommons.org/licenses/by/4.0/ This content is distributed under the terms of the Creative Commons Attribution 4.0 International license.

10.1128/mBio.01590-21.5 FIG S5 NTD loops affect spike incorporation into pseudoviral particles (PPs). The PPs were purified by centrifugation and evaluated by Western blotting. Uncleaved S (S-unc), S2, and VSV M are shown. Download FIG S5, EPS file, 0.4 MB. Copyright © 2021 Qing et al. 2021 Qing et al. https://creativecommons.org/licenses/by/4.0/ This content is distributed under the terms of the Creative Commons Attribution 4.0 International license.

## MATERIALS AND METHODS

### Plasmids.

Full-length SARS-CoV S (GenBank accession no. AY278741.1) and SARS-CoV-2 S, E, M, and N (GenBank accession no. NC_045512.2) genes were synthesized by GenScript, Inc., as human-codon-optimized cDNAs and inserted into pcDNA3.1 expression vectors. C9-tagged versions of the S genes were generated by replacing the 19 3′-terminal codons with linker and C9 codons (GSSGGSSG-GGTETSQVAPA). HiBiT-N was constructed by fusing HiBiT peptide (VSGWRLFKKIS) coding sequences with a linker (GSSGGSSG) to the 5′ end of the N gene, as described in references 42 and 43. The pCMV-LgBiT expression plasmid was purchased from Promega. pDSP_1–7_ and pDSP_8–11_ plasmid DNAs (73, 74) were provided by Zene Matsuda (University of Tokyo). pcDNA3.1-hACE2-C9 was obtained from Michael Farzan, Scripps Florida. pCAGGS-hTMPRSS2_FLAG_ was constructed previously (75). pcDNA3.1-hACE2-LgBiT was constructed by fusing the coding sequence of LgBiT to the 3′ end of hACE2 gene.

### Cells.

HEK293T, HeLa, and HeLa-hACE2 (obtained from Ed Campbell, Loyola University Chicago) cells were maintained in Dulbecco’s modified Eagle medium (DMEM)-10% fetal bovine serum (FBS) (containing 10 mM HEPES, 100 nM sodium pyruvate, 0.1 mM nonessential amino acids, 100 U/ml penicillin G, and 100 μg/ml streptomycin and supplemented with 10% FBS; Atlanta Biologicals). Calu-3 cells (obtained from Paul McCray, University of Iowa) were maintained in MEM-20% FBS (MEM supplemented with 20% FBS, 100 U/ml penicillin G, and 100 μg/ml streptomycin). All cell lines were cultured in a 5% CO_2_ incubator at 37°C.

### Western blotting and antibodies.

Samples in SDS solubilizer (0.0625 M Tris·HCl [pH 6.8], 10% glycerol, 0.01% bromophenol blue, and 2% [wt/vol] SDS with and without 2% 2-mercaptoethanol) were heated at 95°C for 5 min, electrophoresed through 8% or 10% (wt/vol) polyacrylamide-SDS gels, transferred to nitrocellulose membranes (Bio-Rad), and incubated with rabbit polyclonal anti-SARS-CoV-2-S1 (SinoBiological; catalog no. 40591-T62), rabbit polyclonal anti-SARS-S2 (no. JH50520001; obtained from Carolyn Machamer, Johns Hopkins University), mouse anti-C9 (EMD Millipore), mouse monoclonal anti-vesicular stomatitis virus (VSV) M (Kerafast; catalog no. EB0011), goat anti-human IgG (sc-2453; Santa Cruz Biotechnologies), rabbit monoclonal anti-hACE2 (Invitrogen; catalog no. MA5-32307), rabbit polyclonal anti-green fluorescent protein (GFP) (obtained from Katherine Knight, Loyola University Chicago), or purified LgBiT-substrate cocktail (Promega). After incubation with appropriate horseradish peroxidase (HRP)-tagged secondary antibodies and chemiluminescent substrate (Thermo Fisher), the blots were imaged and processed with a FluorChem E apparatus (Protein Simple).

### VLPs.

HiBiT-N-tagged virus-like particles (VLPs) were produced as described previously (42, 43). Briefly, equimolar amounts of full-length CoV S, E (envelope), M (membrane), and HiBiT-N-encoding plasmids (total, 10 μg) were LipoD (SignaGen Laboratories) transfected into 10^7^ HEK293T cells. To produce spikeless (“no-S”) VLPs, the S expression plasmids were replaced with empty vector plasmids. At 6 h posttransfection, cells were replenished with fresh DMEM-10% FBS. HiBiT-N VLPs were collected in FBS-free DMEM from 24 to 48 h posttransfection. FBS-free DMEM containing HiBiT-N VLPs were clarified by centrifugation (300 × *g*, 4°C, 10 min; 3,000 × *g*, 4°C, 10 min).

To obtain purified viral particles, clarified VLP-containing FBS-free DMEM was concentrated 100-fold by ultrafiltration (Amicon; 100 kDa) and then VLPs were purified using size exclusion chromatography (SEC) (original qEV column, used according to product instructions; Izon, Inc.). VLPs were eluted from columns into 2× FBS-free DMEM plus 0.2% FBS. Peak VLP fractions were identified after detergent lysis of VLPs by adding LgBiT and measuring complemented nanoluciferase (Nluc) in a luminometer. Peak fractions were stored at −80°C. Alternatively, VLP-containing FBS-free DMEM samples were overlaid onto 20%, wt/wt, sucrose cushions and particles purified via slow-speed pelleting (SW28, 6,500 rpm, 4°C, 24 h). The resulting pellet was resuspended in FBS-free DMEM to 1/100 of the original medium volumes. SEC peak fractions and resuspended pellets were stored at −80°C.

### VLP cell entry assay.

HeLa target cells were LipoD transfected with pcDNA3.1-hACE2-LgBiT and pCAGGS-TMPRSS2_FLAG_. At 2 days posttransfection, cells were incubated with a live-cell Nluc substrate (Nano-Glo Endurazine; Promega), and 2 h later, HiBiT-N VLPs were inoculated at equivalent HiBiT input multiplicities. HiBiT-N VLPs lacking S proteins (no S) served as negative controls. At hourly intervals following VLP inoculation, Nluc levels were quantified using a Veritas microplate luminometer. For data presentation, the Nluc recordings in cultures inoculated with spikeless (no-S) VLPs were normalized to values of 1.0, and the fold increases over this control condition were calculated and plotted as “relative entry.”

### Cell-free fusion assay.

Cell-free fusion assays required ACE2-LgBiT EVs. To obtain these EVs, HEK293T target cells were LipoD transfected with pcDNA3.1-hACE2-LgBiT. At 6 h posttransfection, transfection media were removed, rinsed, and replaced with FBS-free DMEM. Media were collected at 48 h posttransfection, clarified (300 × *g*, 4°C, 10 min; 3,000 × *g*, 4°C, 10 min), and concentrated 100-fold by ultrafiltration (Amicon; 100 kDa). EVs were then purified using SEC (qEV original; Izon, Inc.) using phosphate-buffered saline (PBS; pH 7.4) as the eluant. Peak EV fractions were identified by the addition of HiBiT-containing detergent and subsequent Nluc measurement by luminometry. EVs were stored at 4°C.

Cell-free fusion assays were performed by mixing HiBiT-N VLPs, each introduced at equivalent HiBiT concentrations, with hACE2-LgBiT EVs, the NanoLuc substrate (catalog no. N2420; Promega), and trypsin (Sigma; 10 ng/μl or as indicated) in 384-well multiwell plates. After 5 min at 4°C, sample plates were loaded into a GloMax luminometer maintained at 37°C. VLP-EV cell-free fusions were quantified as Nluc accumulations over time. For data presentation, the Nluc recordings from samples containing control spikeless (no-S) VLPs were normalized to values of 1.0, and the fold increases over levels for this control condition were calculated and plotted as fold fusion.

For experiments involving S-binding fusion inhibitors, VLPs were premixed at 4°C with serial dilutions of either hACE2:Fc or antibody 576.4 (anti-RBD-antibody, obtained from Hans-Martin Jäck, Friedrich-Alexander-Universität) and then incubated for 30 min at 37°C before the addition of EVs, the substrate, and trypsin. For thermal inactivation experiments, VLPs were preincubated for 0, 24, or 41 h at 37°C, before being mixed with EVs, the substrate, and trypsin at 4°C.

### Pseudoviruses.

Pseudovirus particles (PPs) were constructed from a VSV platform, as described in reference 76. Briefly, HEK293T cells were LipoD transfected for 6 h with S-C9 tag expression plasmids and then replenished with fresh DMEM-10% FBS. At 1 day posttransfection, cells were inoculated for 2 h with VSVdeltaG/Junin GP-luciferase (VSV-luc PP [42, 77]), rinsed extensively, and then replenished with DMEM-10% FBS. Conditioned media were collected 2, 3, and 4 days posttransfection, debris was removed by centrifugation (300 × *g*, 4°C, 10 min; 3,000 × *g*, 4°C, 10 min), and then PPs were pelleted through 20%, wt/wt, sucrose cushions (SW28, 6,500 rpm, 4°C, 24 h) and resuspended in FBS-free DMEM to 1/100 of the original medium volumes. Concentrated PP stocks were stored at −80°C.

### Pseudovirus entry assays.

VSV PPs were inoculated onto Calu-3 cells for 6 h with or without Fc constructs, rinsed extensively, and replenished with FBS-containing DMEM or MEM. At 16 h postransduction, cells were dissolved in lysis buffer (25 mM Tris-phosphate [pH 7.8], 2 mM dithiothreitol [DTT], 2 mM 1,2-diaminocyclohexane-*N,N,N*′-tetraacetic acid, 10% [vol/vol] glycerol, 1% Triton X-100) and mixed 1:2 with a firefly luciferase (Fluc) substrate (1 mM d-luciferin, 3 mM ATP, 15 mM MgSO_4_·H_2_O, 30 mM HEPES [pH 7.8]). Emitted relative light units (RLU) were quantified with a Veritas microplate luminometer.

### Fc constructs.

pCEP4-mCEACAM:Fc was constructed previously (78). Additional constructs were generated using the strategy described in reference 42. Briefly, the mCEACAM coding region was removed by NotI and MreI digestion and replaced with the SARS-CoV-2 S NTD (codons 1 to 309), SARS-CoV-2 NTD-2/1, SARS-CoV-2 S RBD (codons 1 to 24 from the hCD5 signal sequence followed by SARS-CoV-2 S codons 310 to 529), or hACE2 ectodomain (codons 1 to 740). The expression plasmids were LipoD transfected into HEK293T cells, and transfected cells were incubated in FBS-free DMEM containing 2% (wt/vol) Cell Boost 5 (HyClone). Conditioned media were collected on days 4 and 7 and clarified free of debris (300 × *g*, 4°C, 10 min; 4,500 × *g*, 4°C, 10 min), and Fc-tagged proteins were then purified using HiTrap protein A high-performance columns (GE Healthcare) according to the manufacturer’s instructions. Purified proteins were dialyzed in PBS (pH 7.4), quantified spectrophotometrically, and stored at −20°C until use.

### Cell-cell fusion assay.

Effector and target cells were prepared as described previously (42). Briefly, effector HeLa cells were cotransfected with pDSP_1–7_ and pcDNA3.1-SARS-CoV-2-S-C9. Control effector cells received pDSP_1–7_ and empty vector plasmids. Target cells (HeLa or HeLa-hACE2) were cotransfected with pDSP_8–11_ and the indicated hACE2- and/or TMPRSS2 -expressing plasmids. At 30 h posttransfection, target cells were suspended and replated into white-walled 96-well plates. Sixteen hours later, a live-cell *Renilla* luciferase (Rluc) substrate (EnduRen; Promega) and the indicated concentrations of Fc proteins were added. After 2 h, suspended effector cells were distributed into the wells. At hourly intervals following the cocultivation of target and effector cells, Rluc levels were quantified using a Veritas microplate luminometer.

### Statistical analysis.

Statistical comparisons were made by the unpaired Student *t* test. Error bars indicate the standard errors (SE) of the data. *P* values of less than 0.05 were considered statistically significant.
